# The Impact of Pancreatic Head Resection on Blood Glucose Homeostasis in Patients with Chronic Pancreatitis

**DOI:** 10.3390/jcm11030663

**Published:** 2022-01-27

**Authors:** Sebastian Hempel, Florian Oehme, Florian Ehehalt, Michele Solimena, Fiona R. Kolbinger, Andreas Bogner, Thilo Welsch, Jürgen Weitz, Marius Distler

**Affiliations:** 1 Department of Visceral, Thoracic and Vascular Surgery, University Hospital and Faculty of Medicine Carl Gustav Carus, Technische Universität Dresden, 01307 Dresden, Germany; 2 Paul Langerhans Institute Dresden, Helmholtz Center Munich, University Hospital and Faculty of Medicine Carl Gustav Carus, Technische Universität Dresden, 01307 Dresden, Germany

**Keywords:** pancreaticoduodenectomy, duodenum-preserving pancreatic head resection, chronic pancreatitis, beta-cell function, diabetes

## Abstract

Background: Chronic pancreatitis (CP) often leads to recurrent pain as well as exocrine and/or endocrine pancreatic insufficiency. This study aimed to investigate the effect of pancreatic head resections on glucose metabolism in patients with CP. Methods: Patients who underwent pylorus-preserving pancreaticoduodenectomy (PPPD), Whipple procedure (cPD), or duodenum-preserving pancreatic head resection (DPPHR) for CP between January 2011 and December 2020 were retrospectively analyzed with regard to markers of pancreatic endocrine function including steady-state beta cell function (%B), insulin resistance (IR), and insulin sensitivity (%S) according to the updated Homeostasis Model Assessment (HOMA2). Results: Out of 141 pancreatic resections for CP, 43 cases including 31 PPPD, 2 cPD and 10 DPPHR, met the inclusion criteria. Preoperatively, six patients (14%) were normoglycemic (NG), 10 patients (23.2%) had impaired glucose tolerance (IGT) and 27 patients (62.8%) had diabetes mellitus (DM). In each subgroup, no significant changes were observed for HOMA2-%B (NG: *p =* 0.57; IGT: *p* = 0.38; DM: *p* = 0.1), HOMA2-IR (NG: *p =* 0.41; IGT: *p* = 0.61; DM: *p* = 0.18) or HOMA2-%S (NG: *p* = 0.44; IGT: *p* = 0.52; DM: *p* = 0.51) 3 and 12 months after surgery, respectively. Conclusion: Pancreatic head resections for CP, including DPPHR and pancreatoduodenectomies, do not significantly affect glucose metabolism within a follow-up period of 12 months.

## 1. Introduction

Chronic pancreatitis (CP) represents a progressive inflammatory disease leading to destruction and fibrosis of pancreatic tissue, which may cause progressive exocrine as well as endocrine insufficiency. Dilation of the main pancreatic duct, e.g., due to pancreaticolithiasis or fibrosis, causes chronic abdominal pain, which reduces patients’ quality of life. Treatment options for CP comprise endoscopic, interventional as well as surgical procedures. In up to 50% of patients, endoscopic or interventional therapy approaches remain unsuccessful, necessitating surgical management in the course of the disease [1]. The most common indication for surgical treatment of CP is chronic refractory abdominal pain. With regard to pain control and postoperative quality of life, all three available methods of surgical resection—duodenum-preserving pancreatic head resection (DPPHR), pylorus-preserving pancreaticoduodenectomy (PPPD), and classic Whipple procedure (cPD)—yield comparably satisfactory results [2,3]. Yet, in comparison to conservative or interventional treatment modalities, surgical resection risks the loss of healthy pancreatic tissue exerting exocrine and endocrine function. Existing evidence, however, suggests that glucose tolerance can change in either direction after partial pancreatic resection. While new-onset diabetes mellitus (DM) has been found to resolve in over 50% of respective pancreatic cancer patients after tumor resection [4,5], non-diabetic patients bear a significant risk of developing glucose intolerance after partial pancreatic resection [6,7]. CP has been identified as a risk factor for postoperative deterioration of pancreatic endocrine function in pancreatic cancer patients [8,9]. The impact of pancreatic resection in CP patients without pancreatic cancer, however, remains to be fully elucidated.

Most existing studies investigating the metabolic consequences of pancreatic resection primarily display the situation in cancer patients. With this study, we aim to contribute evidence on the effects of different types of pancreatic head resection on blood glucose metabolism in patients with chronic pancreatitis within 12 months after surgery.

## 2. Methods

### 2.1. Study Design and Patient Data Acquisition

This study was designed as a retrospective observational study evaluating prospectively collected data. All patients who underwent PPPD, cPD or DPPHR for chronic pancreatitis between January 2011 and December 2020 at the University Hospital Carl Gustav Carus Dresden, Germany, were screened for eligibility. Only patients with complete follow-up comprising fasting blood tests and oral glucose tolerance tests (OGTT) preoperatively and 3 months and 12 months after surgery were included in this analysis. Blood analysis was prospectively carried out within the OGTT study, in which all analyzed individuals participated based on their informed consent. The OGTT study was approved by the local ethics committee of the TU Dresden (decision number EK 151062008). Medical records including pre- and postoperative data as well as follow-up data for each case were obtained from a prospective database and retrospectively analyzed. The experimental protocol of this retrospective analysis was approved by the local ethics committee of the TU Dresden (decision number BO-EK-480112020). All methods were carried out in accordance with relevant guidelines, and written informed consent was obtained from all included patients.

### 2.2. Surgical Procedures

For CP, we routinely performed pancreatic head resection as PPPD/cPD. In cases of malign inflammatory adhesion to the portal vein, maybe with concomitant occlusion of the portal axis and extended venous collateralization, we preferred DPPHR. The duodenum-preserving procedure was performed as the Berne modification described by Gloor et al. [10].

### 2.3. Blood Glucose Homeostasis

All laboratory tests were performed as fasting blood tests preoperatively as well as 3 and 12 months after surgery during regular outpatient consultations. The following parameters were determined: fasting blood glucose, HbA1c, C-peptide, insulin and proinsulin. Additionally, we performed a 75 g OGTT with all patients who had no DM diagnosed before surgery. According to the American Diabetes Association (ADA) definition [11], patients were stratified as follows: normoglycemic (NG), impaired glucose tolerance (IGT) and diabetes mellitus (DM). The presence of patients with Type 1 DM was excluded by routine measurement of autoantibodies directed against islet autoantigens, as described in Solimena et al. and Winkler et al. [12,13]. Markers of pancreatic endocrine function including steady-state beta cell function (%B), insulin resistance (IR), and insulin sensitivity (%S) were determined according to the updated Homeostasis Model Assessment (HOMA2) [14] using the homacalculator (https://www.dtu.ox.ac.uk/homacalculator/index.php, accessed on 23 August 2021).

### 2.4. Study Endpoints

The primary aim of this study was to evaluate the impact of all types of pancreatic head resection on glucose metabolism. To this end, we investigated global glucose metabolism within the subgroups by examining fasting blood glucose as well as HbA1c values. In order to gain further insights into the remaining beta cell function, insulin resistance and insulin sensitivity after surgery, the corresponding HOMA values within the subgroups were determined and visualized.

### 2.5. Statistical Analysis

For comparison of categorical and quantitative variables, Fisher’s exact test and two-tailed unpaired t-test were used, respectively. A *p*-value < 0.05 was considered statistically significant. Categorical data are expressed as patient number and percentage of the respective patient cohort; quantitative variables are reported as median and interquartile range (IQR). ANOVA analysis was used to compare clinical characteristics and blood sugar homeostasis among the groups (NG, IGT and DM) and among timepoints (preoperative, 3 and 12 months postoperative). The analysis of the HOMA values was performed using generalized linear models and a Bonferroni correction (ANOVA with repeated measurements) was introduced. Mauchly’s sphericity was tested accordingly before the analysis. For data visualization, the IBM SPSS 25 (SPSS Statistics v25, IBM Corporation, Armonk, New York, NY, USA) software package and Microsoft Office 2019 (Microsoft Corporation, Redmond, Washington, DC, USA) were used.

## 3. Results

### 3.1. Baseline Patient Characteristics

**Figure 1:**
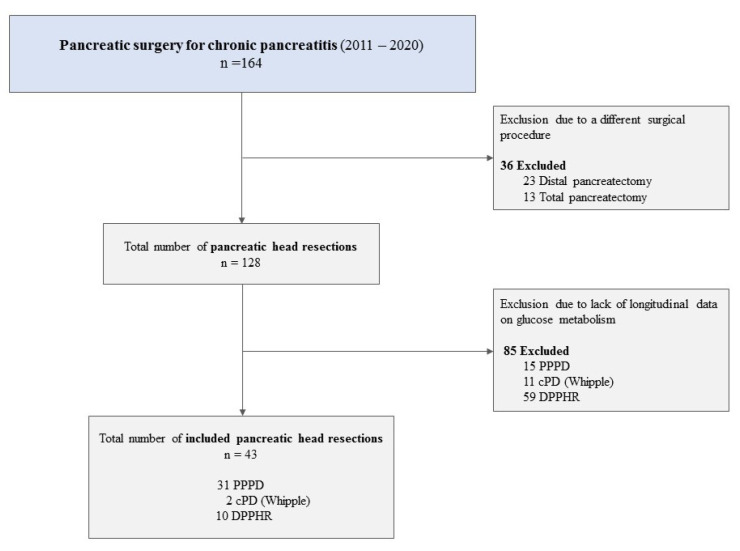
Cases included in and excluded from the study. Abbreviations: cPD, classic pancreatico-duodenectomy (Whipple procedure); DPPHR, duodenum-preserving pancreatic head resection; PPPD, pylorus-preserving pancreaticoduodenectomy.

In total, 164 pancreatic resections were performed for CP within the study period. Out of them, 43 cases of pancreatic head resections including 31 PPPD, 2 cPD und 10 DPPHR met the inclusion criteria (Figure 1).

For further analysis, the patient cohort was stratified into the three subgroups NG (6 patients, 14%), IGT (10 patients, 23.2%), and DM (27 patients, 62.8%). Most included patients were men (*n* = 38, 88.6%). The median age was 53 (IQR 47–57) and the median BMI was 22.2 kg/m^2^ (IQR 20.7–25). The most frequently performed surgery type was PPPD (*n* = 31, 72.1%). Ten patients (23.2%) underwent DPPHR and 2 patients (4.7%) underwent cPD. Relevant postoperative complications (CDC > 2) were observed in 6 cases (14%). The overall median length of hospital stays was 13 days (IQR 12–17). Table 1 displays all baseline patient characteristics.

### 3.2. Impact of Pancreatic Resection on Blood Glucose Homeostasis in Normoglycemic Patients

Patients in the NG group were preoperatively characterized by a normal median fasting glucose of 5.25 mmol/L (IQR 4.65 mmol/L–5.36 mmol/L) and a median HbA1c of 5.2% (IQR 5.1%–5.4%). Three and 12 months after surgery, neither fasting glucose levels (*p* = 0.36) nor HbA1c (*p* = 0.64) changed significantly compared to preoperative levels.

**Figure 2:**
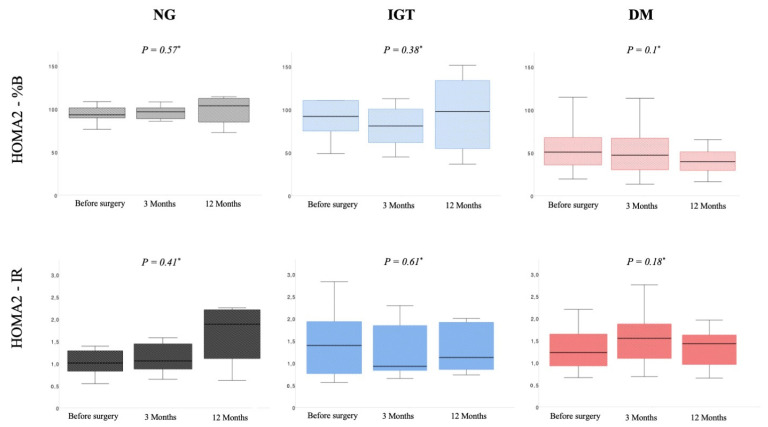
Changes in beta cell function (HOMA2-%B) and insulin resistance (HOMA2-IR) within the three subgroups (NG: *n* = 6, IGT: *n* = 10, DM: *n* = 27) after pancreatic head resection in a 12-month follow-up. * *p*-value refers to analysis across all timepoints. Abbreviations: DM, diabetes mellitus; HOMA2, updated Homeostasis Model Assessment; IGT, impaired glucose tolerance; NG, normoglycemic.

**Figure 3:**
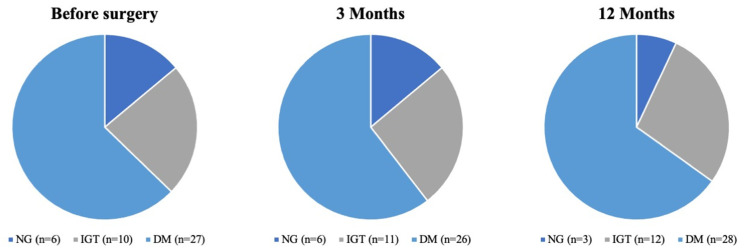
Overall changes in metabolic states within the entire cohort after pancreatic head resection within a 12-month follow-up. Abbreviations: DM, diabetes mellitus; IGT, impaired glucose tolerance; NG, normoglycemic.

Median beta cell function was also not significantly different between the three points of data collection (HOMA2-%B: 91.9% vs. 93.1% vs. 109.0%; *p* = 0.57). Insulin sensitivity (HOMA2-%S) and insulin resistance (HOMA2-IR) were significantly different between the timepoints. Out of the six patients in this group, two subjects developed IGT and one subject developed manifest DM 12 months after surgery. Detailed data are shown in Table 2 and Figure 2. Figure 3 summarizes the distribution of the different metabolic states in the entire cohort.

### 3.3. Impact of Pancreatic Resection on Blood Glucose Homeostasis in Patients with IGT

Table 3 and Figure 2 represent the observed metabolic function parameters in patients with IGT. Changes in median fasting glucose were significant (preoperative median: 5.45 mmol/L (IQR 4.96 mmol/L–6.04 mmol/L) vs. median 12 months postoperative: 5.22 mmol/L (IQR 4.9 mmol/L–6.41 mmol/L), *p* = 0.02), but not clinically relevant. All other parameters were not significantly different between timepoints. In particular, median HOMA2-%B (*p* = 0.38), median HOMA2-%S (*p* = 0.52) and median HOMA2-IR (*p* = 0.61) were not affected by the surgical procedure. Two out of 10 patients changed their glycemic status, developing DM 12 months after surgery.

### 3.4. Impact of Pancreatic Resection on Blood Glucose Homeostasis in Diabetic Patients

The patients in the DM group showed a stable fasting glucose over 12 months of postoperative follow-up (*p* = 0.11). Median HbA1c did not change significantly during the follow-up time from 6.8% (IQR 6.4–7.9%) before surgery, 6.9% (IQR 6.3–7.7%) three months after surgery and 7.4% (IQR 6.7–8.3%) 12 months postoperatively (*p* = 0.36). Beta cell function (HOMA2-%B, *p* = 0.10), insulin resistance (HOMA2-IR, *p* = 0.18) and insulin sensitivity (HOMA2-%S, *p* = 0.51) did not vary significantly between the timepoints. Out of 27 patients in this group, two patients improved their glycemic status to IGT. All other patients remained diabetic. All data are shown in Table 4.

## 4. Discussion

According to the 2016 mechanistic definition, CP describes a continuously progressive pathologic fibro-inflammatory syndrome of the pancreas in individuals with genetic, environmental and/or other risk factors who develop persistent pathologic responses to parenchymal injury or stress [15]. Fibrosis and atrophy of islet cells caused by chronic inflammatory processes result in loss of beta cell function and deterioration of the glycemic status [16].

While conservative and endoscopic treatment options exist, surgical treatment is significantly more cost-effective and, especially with regard to pain relief, superior to non-surgical management of CP [17]. A retrospective analysis of the CP cohort treated at our hospital underlines that surgical therapy should be considered at early disease stages [18]. Moreover, the randomized ESCAPE trial published in 2020 as well as a recent publication by Cahen et al. demonstrate the superiority of early surgical therapy on patients with CP [19,20]. Early surgery could therefore potentially contribute to prevention of deterioration of beta cell function in the course of CP. The optimal surgical approach remains a clinical challenge, since the course of CP is unpredictable. Today, accepted surgical options for treating CP are pancreatic head resections such as modified (Beger/Berne) DPPHR, PPPD, cPD or procedures coring out the (diseased) pancreatic head like the Frey procedure. With regard to quality of life, all mentioned options are equivalent within 24 months after surgery [2]. However, the parenchymal loss inherent to pancreatic surgery can cause postoperative limitations in exocrine and endocrine function. The reported incidence of endocrine insufficiency and new-onset DM after pancreatic resection for CP varies widely in literature due to the retrospective character of studies, different follow-up durations and both heterogeneous cohorts and varying operative techniques.

A recent study by Kumar et al. reported no change in islet function and insulin resistance after the Frey procedure in patients with CP, which is in line with our findings [21]. Still, a trend toward increasing insulin resistance was observed in our normoglycemic group at the 12-month follow-up. In our study, the beta cell function measured by HOMA2-%B did not significantly change 12 months postoperatively compared to preoperative measurements in any of the three metabolic subgroups (NG, IGT, DM). Two previous studies prospectively investigated the impact of the Frey procedure on beta cell function. Izbicki et al. reported postoperative deterioration of C-peptide and serum insulin levels in 14% of cases with preoperatively normal glucose tolerance [22]. One patient out of 4 patients without preoperative DM in this study developed a prediabetic glucose metabolism after surgery. In another study, two and three out of 10 non-diabetic CP patients became prediabetic after the Frey procedure and after PPPD in the 2-year follow-up period, respectively [23]. These findings are in line with our results; however they were gathered in the context of different surgical procedures and follow-up periods.

To the best of our knowledge, there are no studies that prospectively investigate in detail the impact of pancreatic head resection on blood glucose homeostasis in patients with chronic pancreatitis. However, there are some studies that compare outcomes after DPPHR and PPPD. In the ChroPac study, new-onset DM rates of 4% (DPPHR) and 5% (PPPD) were reported at the 2-year follow-up [2]. In contrast, Keck et al. reported incidences of de novo DM as high as 19% and 24% after PPPD and DPPHR, respectively, with a median follow-up duration of 66 months [24]. Comparing the metabolic long-term outcome between the Beger procedure and PPPD, Müller et al. observed no significant differences [25].

Taken together, both the presented analysis and previous evidence suggest comparable results of these surgical procedures with regard to the endocrine long-term outcome. We identified no significant changes, but only trends toward declining beta-cell function and increasing insulin resistance in our cohort regardless of preoperative metabolic state in the 12-month follow-up. In contrast, previous studies suggest that development of new-onset glucose intolerance represents a relevant problem in patients undergoing surgery for CP. Therefore, the impact of different surgical techniques on endocrine pancreatic function in patients with CP remains to be fully elucidated. 

The major limitations of this study are its retrospective character and heterogeneity regarding surgical procedure, number of patients within the subgroups and preoperative disease duration. Furthermore, we do not provide any data on quality of life or exocrine function or on the development of endocrine function after conservative or interventional treatment. Additionally, data from continuous glucose monitors to determine glucose variability would be valuable. However, at this stage we also cannot provide this data. Although these factors are relevant in the overall management of CP, this study focuses on endocrine outcomes of CP combined with pancreatic surgery. Furthermore, the small sample size, especially in the normoglycemic subgroup, is likely underpowered to draw generalizable conclusions. Because of the small cohort size, the comparison between surgical techniques does not yield any meaningful conclusions. Yet, there are comparable studies that report similar results from other perspectives. Given that there is, to our knowledge, no study investigating in detail the impact of pancreatic head resection on blood glucose homeostasis over a 12-month follow-up period, our study adds valuable evidence despite the small cohort size.

## 5. Conclusions

In summary, our study provides evidence that in patients undergoing pancreatic head resection for CP, changes in glucose metabolism within the first year after surgery are rare. However, some patients, particularly those who have already been classified as IGT, are at risk of developing new-onset diabetes within a year postoperatively. Early identification of patients with CP who are at risk of metabolic deterioration could help to provide intensified multifactorial care early on. Besides avoidance of noxious agents, timely surgical therapy to prevent further exocrine and endocrine dysfunction should be considered. In-depth research is needed to fully unravel the exocrine and endocrine consequences of different treatment options for CP.

## Figures and Tables

**Table 1:** Preoperative baseline patient characteristics.

Variable	Overall *n* = 43	NG *n* = 6	IGT *n* = 10	DM *n* = 27	*p*-Value ^1^
Male sex [*n*/(%)]	38 (88.6)	5 (83.3)	8 (80)	25 (92.6)	0.52
Median age [years] (IQR)	53 (47–57)	46 (43–51)	56 (51–64)	53 (49–56)	**0.03**^#,^*
BMI [kg/m^2^] (IQR)	22.2 (20.7–25)	21.5 (21.3–23.5)	22.1 (20.7–23.5)	22.4 (19.6–24.8)	0.98 ^#^
ASA score					0.43
I	1 (2.3)	-	1 (10)	-	
II	23 (53.5)	4 (66.7)	5 (50)	14 (51.9)	
III	19 (44.2)	2 (33.3)	4 (40)	13 (48.1)	
Alcohol abuse [*n*/(%)]	35 (81.4)	6 (100)	8 (80)	21 (77.8)	0.45
Nicotine abuse [*n*/(%)]	34 (79)	6 (100)	7 (70)	21 (77.8)	0.35
Hypertension [*n*/(%)]	21 (48.8)	1 (16.7)	5 (50)	15 (55.6)	0.23
Exocrine insufficiency [*n*/(%)]	14 (32.6)	2 (33.3)	1 (10)	11 (40.7)	0.21
Type of surgery [*n*/(%)]					0.5
*PPPD*	31 (72.1)	5 (83.3)	9 (90)	17 (63)	
*cPD*	2 (4.7)			2 (7.4)	
*DPPHR*	10 (23.2)	1 (16.7)	1 (10)	8 (29.6)	
Length of hospital stay [days] (IQR)	13 (12–17)	13 (12–13)	15 (13–21)	13 (12–16)	0.06 ^#^
Complications CDC > 2 [*n*/(%)]	6 (14)	1 (16.7)	3 (30)	2 (7.4)	0.21

*p*-value refers to analysis across all groups (unless stated otherwise); ^1^ Fisher’s exact test; ^#^ ANOVA; * significance occurred between NG/IGT. Abbreviations: ASA, American Society of Anesthesiologists; BMI, body mass index; CDC, Clavien-Dindo classification; cPD, classic pancreaticoduodenectomy; DM, diabetes mellitus; DPPHR, duodenum-preserving pancreatic head resection; IGT, impaired glucose tolerance; IQR, interquartile range; NG: normoglycemic; PPPD, pylorus-preserving pancreaticoduodenectomy.

**Table 2:** Impact of pancreatic resection on blood glucose homeostasis in normoglycemic patients (*n* = 6).

Variable	Before Surgery	3 Months	12 Months	*p*-Value *
BMI kg/m^2^ (IQR)	21.5 (21.3–23.5)	23.2 (22.1–24.4)	23 (20.9–23.9)	0.21
Fasting glucose mmol/L (IQR)	5.25 (4.65–5.36)	5.29 (5.23–5.42)	5.76 (5.43–5.87)	0.36
HbA1c % (IQR)	5.2 (5.1 -5.4)	5.7 (5.6–5.8)	5.5 (5.2–6.0)	0.64
Insulin nmol/L (IQR)	0.03 (0.02–0.05)	0.03 (0.03–0.04)	0.03 (0.01–0.05)	0.58
C-peptide nmol/L (IQR)	0.44 (0.28–0.48)	0.5 (0.41–0.68)	0.85 (0.59–0.99)	0.27
HOMA2-%B (IQR)	91.9 (88.1–100.4)	93.1 (82.8–98.8)	109 (93.8–118)	0.57
HOMA2-%S (IQR)	93.5 (74–113.5)	89.5 (65.1–108.7)	52.8 (43.8–94.6)	0.44
HOMA2-IR (IQR)	1.07 (0.88–1.55)	1.12 (0.92–1.54)	1.95 (1.35–2.28)	0.41

* One-way analysis of variance (ANOVA) with repeated measurements, *p*-value refers to analysis across all timepoints. Abbreviations: BMI, body mass index; HOMA2, updated Homeostasis Model Assessment; IQR, interquartile range.

**Table 3:** Impact of pancreatic resection on blood glucose homeostasis in patients with IGT (*n* = 10).

Variable	Before Surgery	3 Months	12 Months	*p*-Value *
BMI kg/m^2^ (IQR)	22.1 (20.7–23.5)	21.3 (20.3–23.2)	22.2 (21–24.4)	**0.04**
Fasting glucose mmol/L (IQR)	5.45 (4.96–6.04)	5.33 (4.95–6.15)	5.22 (4.9–6.41)	**0.02**
HbA1c % (IQR)	5.5 (5.4–6.0)	5.9 (5.3–6.3)	6.0 (5.8–6.3)	0.69
Insulin nmol/L (IQR)	0.04 (0.02–0.07)	0.03 (0.01–0.06)	0.03 (0.02–0.05)	0.64
C-peptide nmol/L (IQR)	0.6 (0.32–0.86)	0.35 (0.29–0.73)	0.54 (0.37–0.83)	0.54
HOMA2-%B (IQR)	91.9 (74.7–108.6)	79.5 (57.8–101.4)	98.6 (51.4–131)	0.38
HOMA2-%S (IQR)	73.9 (50.2–138.4)	120 (52.4–139.1)	87.1 (48.4–118.4)	0.52
HOMA2-IR(IQR)	1.4 (0.7–2)	0.8 (0.7–1.9)	1.2 (0.8–2.1)	0.61

* One-way analysis of variance (ANOVA) with repeated measurements, *p*-value refers to analysis across all timepoints. Abbreviations: BMI, body mass index; HOMA2, updated Homeostasis Model Assessment; IQR, interquartile range.

**Table 4:** Impact of pancreatic resection on blood glucose homeostasis in diabetic patients (*n* = 27).

Variable	Before Surgery	3 Months	12 Months	*p*-Value *
BMI kg/m^2^ (IQR)	22.5 (19.9–25.7)	22.9 (20.8–25.6)	23.3 (21.2–25.3)	0.16
Fasting glucose mmol/L (IQR)	7.79 (6.27–8.54)	7.77 (6.62–10.29)	7.78 (6.34–9.74)	0.11
HbA1c % (IQR)	6.8 (6.4–7.9)	6.9 (6.3–7.7)	7.4 (6.7–8.3)	0.36
Insulin nmol/L (IQR)	0.03 (0.01–0.07)	0.03 (0.01–0.05)	0.02 (0.01–0.04)	0.18
C-peptide nmol/L (IQR)	0.38 (0.18–0.58)	0.45 (0.17–0.65)	0.33 (0.16–0.59)	0.22
HOMA2-%B (IQR)	50.3 (34.5–68.3)	49.3 (31.2–70.4)	39.8 (28.8–52.6)	0.1
HOMA2-%S (IQR)	81.4 (59.8–111.4)	61.3 (49.8–91.1)	66.4 (57.5–104.2)	0.51
HOMA2-IR (IQR)	1.23 (0.9–1.69)	1.63 (1.1–2.01)	1.39 (0.96–1.74)	0.18

* One-way analysis of variance (ANOVA) with repeated measurements, *p*-value refers to analysis across all timepoints. Abbreviations: BMI, body mass index; HOMA2, updated Homeostasis Model Assessment; IQR, interquartile range.

## Data Availability

The data are available from the corresponding author on request.

## Abbreviations

ADA	American Diabetes Association
BMI	body mass index
CDC	Clavien-Dindo classification
cPD	classic pancreaticoduodenectomy
CP	chronic pancreatitis
DM	diabetes mellitus
DPPHR	duodenum-preserving pancreatic head resection
HOMA2	updated Homeostasis Model Assessment
IGT	impaired glucose tolerance
IQR	interquartile range
NG	normoglycemic
OGTT	oral glucose tolerance test
PPPD	pylorus-preserving pancreaticoduodenectomy
